# Validation of a reversed-phase high-performance liquid chromatography method for quantification of allantoin, creatinine, and uric acid in individual spot cow urine samples

**DOI:** 10.3168/jdsc.2025-0855

**Published:** 2025-10-10

**Authors:** E. Visentin, I. Sousa, S. Magro, S. Sabbadin, G. Niero, M. De Marchi

**Affiliations:** Department of Agronomy, Food, Natural resources, Animals and Environment, University of Padova, Viale dell'Università 16, 35020 Legnaro (PD), Italy

## Abstract

The possibility of monitoring allantoin, creatinine, and uric acid in individual cow urine can provide insights into the physiological responses of dairy cows under heat stress conditions and offer information on nitrogen metabolism. The aim of this study was to validate a reversed-phase HPLC method for the simultaneous quantification of allantoin, creatinine, and uric acid in dairy cow urine. Urine samples were collected from 10 lactating Holstein cows and were analyzed in triplicate for 5 consecutive days, for an overall total of 150 analyses. The method demonstrated good repeatability and reproducibility performances. In particular, repeatability coefficients exceeded 99% for allantoin and creatinine, and surpassed 95% for uric acid. Among the compounds analyzed in the present study, creatinine and uric acid exhibited the highest and the lowest coefficient of reproducibility (99.39% and 78.03%, respectively). The method exhibited optimal linearity for dilutions between 1:20 (i.e., 1 part of urine and 20 parts of water) and 1:8 (i.e., 1 part of urine and 8 parts of water), with R^2^ ranging from 0.98 to 0.99 for uric acid and allantoin, respectively. The best recovery rates were obtained at the highest spike level for allantoin (102.97%), at the medium spike level for creatinine (98.70%), and at the lowest spike level for uric acid (95.61%). This study may lay the groundwork for future research on the association between novel urine biomarkers and temperature and humidity indexes.

## Highlights

• Allantoin, creatinine, and uric acid were quantified in individual spot cow urine.
• High repeatability and reproducibility were achieved.
• Robust linearity was found across the tested dilution ranges.
• High recovery rates were reached for allantoin, creatinine, and uric acid.

## Graphical Abstract

**Figure:**
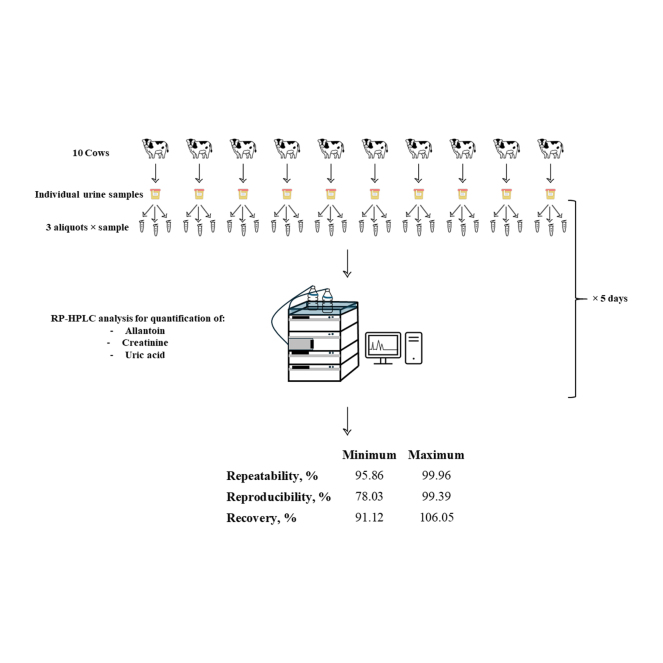
**Summary:** The aim of this study was to validate a chromatographic method to detect and quantify allantoin, creatinine, and uric acid in cow urine samples. The method featured a simple chromatographic separation, yielding well-defined peaks. High repeatability and reproducibility were achieved, with optimal linearity performances. Excellent recovery rates were obtained, especially for allantoin and creatinine. The findings of this study confirm the effectiveness of the method in analyzing target compounds, laying the foundation for a better understanding of physiological responses to heat stress, one of the major challenges facing the future of the livestock sector.

Cow urine represents a potentially informative matrix for biological screening, and may complement analyses based on other substrates such as milk and blood. It may also offer insights into physiological responses under heat stress conditions, influencing nitrogen metabolism. In particular, urinary allantoin, creatinine, and uric acid serve as indirect markers of rumen microbial protein synthesis and nutritional efficiency, which are closely linked to feed intake (Reynal et al., 2005). Indeed, a reduction in feed intake leads to decreased microbial activity in the rumen, which secondarily alters urinary concentrations of allantoin, creatinine, and uric acid (González-Ronquillo et al., 2004). Allantoin is a product of uric acid oxidation. A critical factor in the conversion of uric acid to allantoin is rumen microbial activity, which is strongly related to diet and stress factors (Giesecke et al., 1994). Compared with uric acid, allantoin is more water-soluble and is typically excreted in the urine by ruminants (Roman, 2023). Allantoin has emerged as a reliable biomarker for various health conditions, including oxidative stress (Liao et al., 2018). Indeed, increased concentrations of allantoin should be expected in the urine of cows experiencing heat stress due to increased oxidative conditions (Liao et al., 2018). Creatinine concentration in urine is affected by several factors, including diet, age, and animal physiological status, such as pregnancy (Herman et al., 2019). The urinary protein-to-creatinine ratio is an informative trait for assessing renal dysfunction in cattle, making it a valuable biomarker for assessing animal health (Katsoulos et al., 2020). Uric acid is a byproduct of protein metabolism, mainly excreted by kidneys. Uric acid is also involved in immune cell activation and acts as a selective antioxidant, helping to counteract oxidative stress. Kim et al. (2024) observed a significant reduction in hematic uric acid of cows exposed to heat stress. This aligns with the findings of Liao et al. (2018), who reported that lower uric acid levels and greater allantoin levels in urine indicate increased oxidative stress.

To date, the determination of allantoin, creatinine, and uric acid have been carried out through diverse chromatographic approaches, in different ruminant species and substrates. For instance, Moscardini et al. (1999) adapted HPLC protocols to simultaneously determine major purine derivatives, including allantoin, uric acid, xanthine, and hypoxanthine in cattle, sheep, buffalo, and camel urine. Czauderna and Kowalczyk (1997) developed an HPLC method for blood plasma analysis in ruminants, enabling simultaneous measurement of oxypurines and allantoin. They later extended this approach to ovine urine in Czauderna and Kowalczyk (2000), developing a photodiode array-based HPLC protocol that achieved complete separation of purine metabolites. Nevertheless, in terms of analytical methodology, the literature on HPLC methods for quantifying allantoin, creatinine, and uric acid in cattle urine remains inconsistent, often providing fragmented and incomplete information on method validation parameters. Shingfield and Offer (1999) proposed an HPLC protocol for the simultaneous detection of allantoin, creatinine, and pseudouridine in ruminant urine, yet they did not provide data on repeatability or reproducibility. Resines et al. (1992) described a reversed-phase HPLC (**RP-HPLC**) method to quantify allantoin and creatinine in sheep urine, focusing on interday bias without offering details on intraday trials. George et al. (2006) reported application of an improved HPLC approach for analyzing allantoin, creatinine, and uric acid in cattle urine, including both intra- and interday variability estimates, but omitted broader validation parameters such as accuracy or robustness. Based on this background, the aim of this study was to assess repeatability, reproducibility, linearity, and recovery of an RP-HPLC method for simultaneous quantification of allantoin, creatinine, and uric acid in individual spot cow urine, thus ensuring a consistent assessment of the key method validation parameters.

The study was exempted from authorization and ethical approval by the animal welfare committee of the University of Padova (Ref. OPBA n. 42/2025), as the procedures were deemed noninvasive. Ultrapure water (18.2 MΩ/cm resistivity at 25°C) was produced through Arium basic (Sartorius Stedium Biotech, Varedo, Italy). Commercial standards of allantoin (lot LRAD3303; ≥98% purity), creatinine (lot LRAC6514; ≥99% purity), and uric acid (lot BCCK0750; ≥99% purity) were purchased from Sigma-Aldrich (St. Louis, MO). Sample analyses were performed through an RP-HPLC Agilent 1260 Infinity II LC system (Agilent Technologies, Santa Clara, CA) equipped with a quaternary pump (Agilent 1260 Infinity II, G7111B), a diode array detector (Agilent 1260 Infinity II, G7115A), and a refrigerated auto-sampler (Agilent 1260 Infinity II, G7129A) able to maintain sample vials at a constant temperature (4°C–40°C operating temperature).

Urine samples were obtained from 10 lactating Holstein cows housed in a single commercial farm located in the Veneto Region (Italy). Cows were fed a TMR consisting of corn silage (19.00 kg/cow per d), mixed hay (2.60 kg/cow per d), ryegrass haylage (3.70 kg/cow per d), straw (1.2 kg/cow per d), soybean meal (3.30 kg/cow per d), vitamin and mineral mix integrator (0.35 kg/cow per d), concentrate supplement (3.00 kg/cow per d), and water (3.00 kg/cow per d). The average chemical composition of the diet on a DM basis was 25.89% starch, 14.22% CP, 2.71% ether starch, 6.16% total ash, 33.54% NDF, 18.36% ADF, and 2.07% ADL. The trial was conducted under environmental conditions of 27.81°C ambient temperature and 61.94% relative humidity. Cows had an average BW of 730.29 kg (minimum: 615.51 kg; maximum: 855.96 kg). Animals were characterized by average DIM of 140.3 d (minimum: 13 d; maximum: 235 d), average parity of 2.3 (minimum: 1 parity; maximum: 4 parities), and average milk yield of 30.30 kg/d (minimum: 19.70 kg/d; maximum: 39.70 kg/d). On d 1, before morning milking, an external bladder massage was performed to induce urination, and individual urine samples were collected into sterile 150-mL plastic containers. After collection, urine samples were immediately transferred to the laboratory of the Breeders Association of Veneto Region (Vicenza, Italy) and mesh-filtered to remove any suspended solids. Each sample was divided into 3 aliquots of 1 mL each (for a total of 30 aliquots), which were successively prepared for RP-HPLC analysis. The same aliquoting procedure was repeated on the following 4 d (i.e., d 2, 3, 4, and 5), resulting in a total of 150 urine aliquots analyzed by RP-HPLC over the 5-d period.

Urine samples were prepared following the method proposed by George et al. (2006). A volume of 1 mL of urine was centrifuged at room temperature for 15 min at 13,000 × *g* to promote particle residues precipitation. Therefore, the supernatant was diluted in ultrapure water (1:10) in disposable plastic tubes. After dilution, samples were gently inverted 10 times and filtered using a 0.22-μm syringe filter. Finally, 1 mL of the filtered solution was transferred into disposable glass vials for RP-HPLC analysis. The RP-HPLC conditions for the separation of allantoin, creatinine, and uric acid were adapted from the method proposed by George et al. (2006). Chromatographic separation was performed using a reversed-phase analytical column C18 (InfinityLab Poroshell 120 EC-C18, Agilent Technologies) with a silica-based packing (4.6 × 250 mm, 4 μm) preceded by a pre-column UHPLC Guard 3PK (InfinityLab Poroshell 120 EC-C18, 4.6 × 5 mm, 4 μm, Agilent Technologies), and applying an isocratic elution based on 10 m*M* potassium dihydrogen phosphate as mobile phase. Flow rate was set at 1 mL/min, column temperature was kept at 25°C, and detection was made at a wavelength of 220 nm. Sample vials were kept refrigerated (5°C) and the injection volume was 20 μL. The total analysis time per sample was 20 min. Agilent OpenLab 2 CDS, version 2.5.0.927 software (Agilent Technologies, Santa Clara, CA) was used for data acquisition and analysis. Each chromatographic peak was identified using internal and external standards. Quantification of allantoin, creatinine, and uric acid was performed through a 5-point calibration curve, specifically developed for each target compound. All calibration curves exhibited an R^2^ greater than 0.99.

Variance components for assessing repeatability and reproducibility of allantoin, creatinine, and uric acid concentration were estimated using the following mixed linear model, implemented in R software v 4.3.3, through the ‘lme4‘ package (R Core Team, 2022):

[1] *y_ij_ = µ* + *Day_i_* + *Cow_j_* + (*Day* × *Cow*)*_ij_* + *e_ij_*,

where *y_ij_* is uric acid, allantoin, and creatinine concentration in individual urine samples; *µ* is the overall intercept of the model; *Day_i_* is the random effect of the *i*th day of analysis (*i* = 1 to 5); *Cow_j_* is the random effect of the *j*th cow (*j* = 1 to 10); (*Day* × *Cow*)*_ij_* is the random interaction effect between the *i*th day of analysis and the *j*th cow; and *e_ij_* is the random error term. All random effects were considered to be independent and normally distributed, with mean zero and proper variance (i.e., $\sigma_{Day}^{2},$ $\sigma_{Cow}^{2},$ $\sigma_{DayxCow}^{2},$ and $\sigma_{e}^{2}$ for day of analysis, cow, their interactions, and residual, respectively). Coefficient of repeatability (**C_RPT_**) was calculated as an indicator of the consistency of allantoin, creatinine, and uric acid concentrations measured in the same urine sample on the same day, according to the following formula (Penasa et al., 2015):

$$C_{\mathrm{RPT}},\;\%=\frac{\sigma_{Cow}^{2}+\sigma_{Day}^{2}+\sigma_{DayxCow}^{2}}{\sigma_{Cow}^{2}+\sigma_{Day}^{2}+\sigma_{DayxCow}^{2}+\sigma_{e}^{2}}\times 100.$$

Coefficient of reproducibility (**C_RPD_**) was calculated as an indicator of the consistency of allantoin, creatinine, and uric acid concentrations measured in the same urine sample on different days, according to the following formula (Penasa et al., 2015):

$$C_{\mathrm{RPD}},\;\%=\frac{\sigma {{}_{Cow}^{2}}_{\;}}{\sigma_{Cow}^{2}+\sigma_{Day}^{2}+\sigma_{DayxCow}^{2}+\sigma_{e}^{2}}\times 100.$$

Linearity of the method was evaluated through the R^2^ obtained from the regression between increasing dilutions of urine in water (i.e., 1:8, 1:10, 1:13, 1:14, 1:17, 1:20) and the resulting concentration of allantoin, creatinine, and uric acid (Figure 1). The lower limit of quantification (**LOQ**) was defined as the lowest calibration point of the regression, whereas the upper LOQ corresponded to the highest calibration point on the same regression.

**Figure 1:**
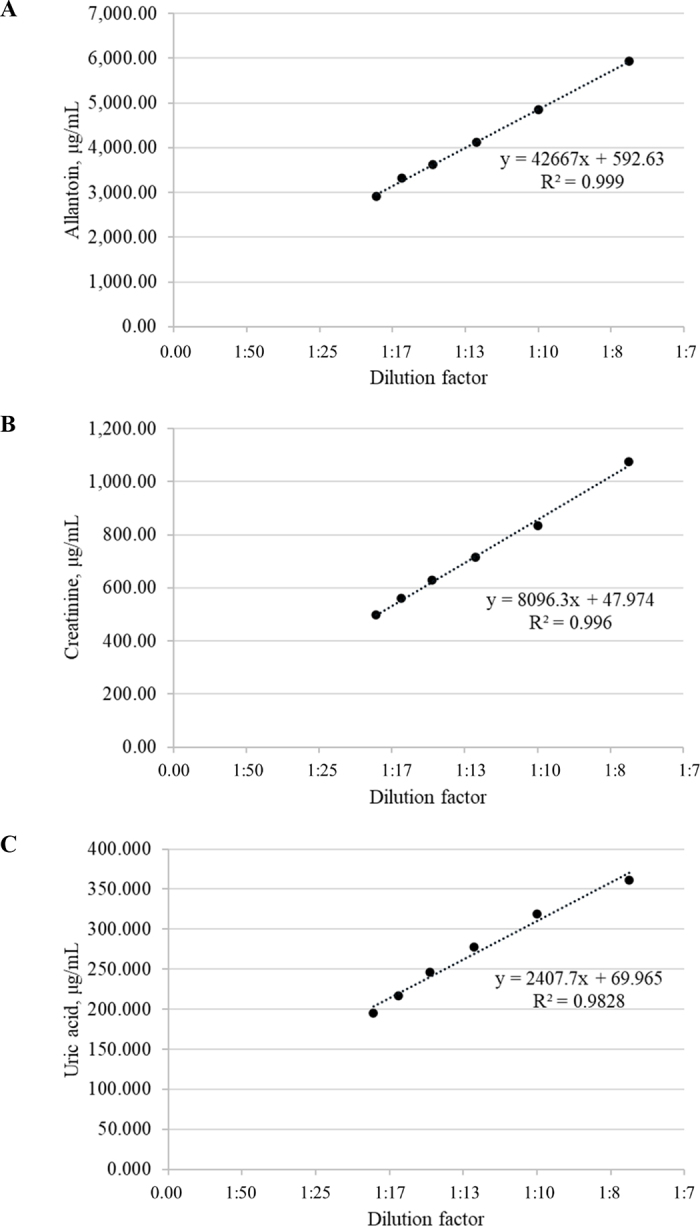
Linearity tests for concentration of allantoin (A), creatinine (B), and uric acid (C), performed on different dilutions of the same urine sample.

Recovery was assessed at 3 spiking levels. Spiking standard mix was prepared by dissolving 40 mg of allantoin, 8 mg of creatinine, and 4 mg of uric acid in 9.3 mL of ultrapure water, with uric acid pre-dissolved in 0.7 mL of a NaOH solution. Three spiking levels were prepared by mixing different proportions of spiking standard mix with urine to a final volume of 1 mL. Specifically, the lowest spike level contained 200 µL of the standard mix in 800 µL of urine (final concentrations of 800 µg/mL for allantoin, 160 µg/mL for creatinine, and 80 µg/mL for uric acid); the medium spike level contained 300 µL of the standard mix in 700 µL of urine (final concentrations of 1,200 µg/mL for allantoin, 240 µg/mL for creatinine, and 120 µg/mL for uric acid); and the highest spike level contained 400 µL of the standard mix in 600 µL of urine (final concentrations of 1,600 µg/mL for allantoin, 320 µg/mL for creatinine, and 160 µg/mL for uric acid). Recovery was calculated as the ratio between the measured concentrations of the spiked sample and the expected concentrations of the same spiked sample (Francisco and Resurreccion, 2009):

$$\mathrm{recovery},\;\%\;=\left(\frac{\mathrm{measured}\;\mathrm{concentration}\;}{\mathrm{expected}\;\mathrm{concentration}}\right)\times 100.$$

For each compound, average recovery and SD were calculated on 2 replicates of the same spike level.

The chromatographic analysis of urine samples revealed 2 major peaks corresponding to creatinine and allantoin, and a smaller third peak related to uric acid. The chromatographic profile demonstrated satisfactory resolution for all peaks, with minimal background noise and no interfering signals, thus providing an efficient separation of the 3 target compounds. The elution order observed in the present study (i.e., allantoin, creatinine, and uric acid) aligns with the findings of George et al. (2006) and Resines et al. (1992) but differs from that reported by Shingfield and Offer (1999), where allantoin eluted first, followed by uric acid and creatinine. In the present trials, allantoin, creatinine, and uric acid had a retention time of 2.16, 2.84, and 3.49 min, respectively, whereas George et al. (2006) reported longer retention time of 3.3 min for allantoin, 4.2 min for creatinine, and 10.2 min for uric acid. Retention times obtained in the present study were shorter than those reported by Resines et al. (1992), who found retention time of 3.9 min for allantoin, 5.7 min for creatinine, and 7.2 min for uric acid. The variations in retention time between our study and George et al. (2006) can be related to (1) pH adjustments that are not required in our method and (2) differences in the chromatographic equipment and apparatus. Instead, the longer retention time observed by Resines et al. (1992) may be attributed to (1) different flow rate (0.50 mL/min, instead of 1.00 mL/min used in the present study), (2) different biological matrix (sheep urine, instead of cow urine of the present study), and (3) different wavelength used for the detection of target compounds (218 nm, instead of 220 nm of the present study).

The stability of the analytical method was assessed by analyzing the concentrations of allantoin, creatinine, and uric acid in individual cow urine samples for 5 consecutive days of analysis. Table 1 shows an increasing trend in allantoin concentrations, from 2,891.15 µg/mL on d 1 to 3,768.99 µg/mL on d 5. These findings are in line with those obtained by Giesecke et al. (1994) and Charteris et al. (2021), who observed that storage significantly affected the concentration of urine nitrogenous compounds, due to microbial and enzymatic activity. The observed variability in respect to the measured concentration of allantoin over the 5 d of analysis did not affect repeatability in terms of C_RPT_ (99.96%), but partially affected reproducibility in terms of C_RPD_ (90.86%; Table 2). Creatinine and uric acid concentrations remained relatively stable along the experimental trials (Table 1). This stability is reflected into optimal C_RPT_ and C_RPD_ for creatinine (C_RPT_ = 99.94%, C_RPD_ = 99.39%) and into satisfactory C_RPT_ and C_RPD_ for uric acid (C_RPT_ = 95.86%, C_RPD_ = 78.03%; Table 2). This is likely due to the relatively low susceptibility of these compounds to microbial degradation, as both creatinine and uric acid are less influenced by enzymatic breakdown compared with allantoin (Charteris et al., 2021). The consistency of creatinine concentration observed in the present study aligns with findings by Danese et al. (2024), who observed that urinary creatinine concentrations in dairy cattle remained stable under controlled storage conditions. Similarly, the stability of uric acid over time observed in this study aligns with the findings of George et al. (2006), who reported that uric acid concentrations in cattle urine remained unchanged across different storage periods and conditions. Although method repeatability and reproducibility were satisfactory, it is important to note that the stability of nitrogen metabolites such as allantoin may be compromised during storage. Even short delays in analysis can lead to substantial alterations in these compounds. da Silva Júnior et al. (2021) emphasized the necessity of immediate cooling and standardized preservation protocols to prevent degradation and ensure accurate measurements in metabolic studies involving urine samples. Similar conclusions were drawn by Magro et al. (2024) in a study on the prediction of milk chemical composition and by Penasa et al. (2015) in a study on milk coagulation properties.

**Table 1:** Average concentration (SD) of allantoin, creatinine, and uric acid (μg/mL) in individual cow urine samples, within and across days of analysis

Trait	Days of analysis					
	d 1 (n = 30)	d 2 (n = 30)	d 3 (n = 30)	d 4 (n = 30)	d 5 (n = 30)	Overall (n = 150)
Allantoin	2,891.15	3,083.43	3,241.03	3,542.38	3,768.99	3,305.40
	(1,204.98)	(1,191.85)	(1,114.85)	(1,134.489)	(1,201.91)	(1,196.88)
Creatinine	596.60	614.55	594.86	594.86	608.79	603.03
	(206.60)	(212.38)	(208.70)	(206.76)	(219.85)	(208.10)
Uric acid	155.55	149.47	151.66	158.04	156.66	154.27
	(21.98)	(17.38)	(16.13)	(20.16)	(23.34)	(19.97)

**Table 2:** Coefficient of repeatability (C_RPT_) and coefficient of reproducibility (C_RPD_) for the concentration of allantoin, creatinine, and uric acid in individual cow urine samples^1^

Trait	C_RPT_	C_RPD_	Low spike^2^	Medium spike^3^	High spike^4^
Allantoin	99.96	90.86	104.36 (0.12)	106.05 (1.60)	102.97 (1.10)
Creatinine	99.94	99.39	97.71 (0.04)	98.70 (0.21)	96.66 (0.40)
Uric acid	95.86	78.03	95.61 (1.61)	94.18 (0.09)	91.12 (2.65)

1 Average recovery (SD) for the concentration of allantoin, creatinine, and uric acid in individual cow urine samples (calculated on 2 replicates).

2 Low spike: 200 μL of the standard mix in 800 μL of urine, resulting in final concentrations of 800 μg/mL for allantoin, 160 μg/mL for creatinine, and 80 μg/mL for uric acid.

3 Medium spike: 300 μL of the standard mix in 700 μL of urine, resulting in final concentrations of 1,200 μg/mL for allantoin, 240 μg/mL for creatinine, and 120 μg/mL for uric acid.

4 High spike: 400 μL of the standard mix in 600 μL of urine, resulting in final concentrations of 1,600 μg/mL for allantoin, 320 μg/mL for creatinine, and 160 μg/mL for uric acid.

Equations obtained from linearity tests are expressed as *y* = *mx* + *b*, where *m* represents the slope and *b* the y-intercept (Figure 1). In the present study, R^2^ calculated for linearity tests exceeded 0.99 for both allantoin and creatinine and was above 0.98 for uric acid (Figure 1). These results indicate optimal linearity performance within the lowest and the highest dilutions tested in the trials of this study (i.e., 1:8 and 1:20, respectively). Moreover, the lower and upper LOQ were determined based on the lowest and highest calibration points included in the regression line, respectively. Specifically, for allantoin, the quantitative range extended from 2,918.60 to 5,927.74 µg/mL; for creatinine, from 499.47 to 1,075.32 µg/mL; and for uric acid, from 194.74 to 361.66 µg/mL.

The accuracy of the method was assessed by measuring the recovery rates of known amounts of allantoin, creatinine, and uric acid added to urine samples (Table 2). Among the compounds analyzed in the present study, allantoin exhibited the best recovery rate at the highest spike level, with a value of 102.97%, indicating minimal deviation from the ideal value of 100%. The lowest variability for the recovery of allantoin was observed at the lowest spike level, with a SD of 0.12%. In contrast, the medium spike level had the greatest SD along with the greatest deviation from 100% (Table 2). In our study, recovery levels for allantoin consistently exceeded 100% across all spiking concentrations. Conversely, George et al. (2006) reported a recovery rate of 100.05% at the highest spiking concentration of 150 µg/mL. This discrepancy may be attributed to the higher concentrations used in our spiking trials compared with those performed by George et al. (2006). Creatinine showed the best recovery for the medium spike level (98.70%; Table 2). The recovery for creatinine improved from the lowest to the medium spike level, although SD increased. As the spiking concentration increased from medium to high levels, creatinine recovery rates decreased and the SD increased, indicating that the highest spiking concentration exhibited the lowest recovery rate. Our results showed lower SD compared with that reported by George et al. (2006) and Shingfield and Offer (1999). This difference may be attributed to the different number of samples analyzed for recovery trials; however, recovery rates reported in the present study for creatinine are similar to those reported by George et al. (2006; 98.7%) and by Shingfield and Offer (1999; 98.77%). Uric acid showed similar recovery rates for the lowest spike level (95.61%) and for the medium spike level (94.18%). Nevertheless, SD for the medium spike level was considerably lower compared with that of the lowest spike level (0.09% and 1.61%, respectively). In general, uric acid presented lower recovery rates compared with allantoin and creatinine. This trend was also observed by Shingfield and Offer (1999), where the average recovery of uric acid (96.9%) was lower than that of allantoin (100.6%) and creatinine (98.77%). In terms of SD, the observed variability suggests that recovery can be influenced by both the nature of specific compounds and the spiking concentration. Both uric acid and creatinine exhibited increased variability in recovery rates at the highest spiking level, indicating that solvents used in sample preparation may have reached saturation. Uric acid, in particular, showed the lowest overall recovery, potentially due to pH fluctuations, which might have led to an underestimation of its concentration (George et al., 2006).

The present RP-HPLC method offers relevant economic and environmental advantages, with practical implications at the dairy sector level. Indeed, minimal sample preparation without consumption of organic solvents and a single 20-min chromatographic run reduce reagent use, operator time, and overall costs, making this method economically and environmentally sustainable. Moreover, the possibility of monitoring allantoin, creatinine, and uric acid in cattle urine opens the possibility of screening animal health and nutrition status at both individual and large-scale levels.

In this study, an RP-HPLC method was employed to determine allantoin, creatinine, and uric acid in dairy cow urine. Satisfactory repeatability and reproducibility values were observed for all compounds, with optimal results for allantoin and creatinine. The method exhibited excellent linearity (R^2^ > 0.98) and recovery rates ranging from 91.12% to 106.05%. Taken together, these results establish the developed RP-HPLC method as an effective and reliable tool for quantifying allantoin, creatinine, and uric acid in cow urine. Importantly, this method represents a versatile assay to support future studies, enabling monitoring of these biomarkers in relation to variables such as heat stress, dietary regimens, and other environmental or physiological influences affecting livestock health.
